# 

**Published:** 1851-11

**Authors:** 


### CONTENTS

### OF NO. V.

### ORIGINAL COMMUNICATIONS.

### ESSAYS AND CASES.

Art. I.—An Important and Dangerous Surgical Operation. By M. Lenoir, at Necker Hospital, Paris. Translated by P. K. - - - - - - - - - 369
Art. II.—Proceedings of a Convention of the Physicians of Kentucky. Reported by D. W. Yandell, M.D., of Louisville, Ky. - - - - - - - - - 372
Art. III.—Practical Medicine. By Theodore S. Bell, M.D. 378
A New Epidemic Exanthem. - - - - - - 378
Remedy for Deficient Lactation. - - - - - - - 382
Yeast in Malignant Scarlet Fever, - - - - 382
Treatment of Small-Pox. - - - - - - - - - 383
Peculiar Appearance in the Gums of Consumptive Patients. 384
Effects of Aneurism. - - - - - - - - - - 386
New method of Reducing Dislocation of the Femur on Dorsum Ilii. - - - - - - - - - - - 391
Ovarian Irritation. - - - - - - - - - 394
Early Viability. - - - - - - - - - - - 396
Canabis Indica as a Substitute for Ergot. - - - 397
Vesico-Vaginal Fistula, Cured by an Operation. - - 398
Malignant Pustule or Carbuncle. - - - - - 401
Treatment of Urticaria by the Sulphate of Quinine. - 402
Pertussis. - - - - - - - - - - - 403
Prophylaxis of Puerperal Fever. - - - - - - - 404

### REVIEWS.

Art IV.—Surgical Anatomy. By Joseph Maclise, Surgeon. With colored plates. - - - - - - - 409

ART. V.—Southern Medical Reports. Edited by E. D. Fenner, M.D., of New Orleans. - - - - - - - 418
ART. VI.—Elements of General and Pathological Anatomy, presenting a view of the present state of knowledge in these branches of Science. By David Craigie, M.D., F.R.S.E. - - - - - - - - - 421
ART. VII.—Special Anatomy and Histology. By Wm. E. Horner, M.D. - - - - - - - - - 422
ART VIII.—The Laws of Health, in relation to mind and body; a series of Letters from an old Practitioner to a Patient. By Lionel John Beale, M.R.C.S. - - - 423

SELECTIONS FROM FOREIGN AND HOME JOURNALS.

Cases of Amputation, - - - - - - - - 429
Vaccination and Small Pox, - - - - - - - 448
Austrian Remedy for Cholera, - - - - - - 449
Use of an Aqueous Solution of Tartar, - - - - - 451
Case of Rape—Death the Consequence, - - - - - 452

ORIGINAL INTELLIGENCE.

Vital Statistics of Fayette County, Ky., - - - - - 453
Nelson's Northern Lancet, and American Journal of Medical Jurisprudence, - - - - - - - - - - - 455
The Health of Louisville, - - - - - - - 456
Medical Lectures, - - - - - - - - - - 456
Professor Austin Flint's Fever Reports, - - - - - 457
Kentucky State Medical Society, - - - - - - - 457
The Honors of War, - - - - - - - - - 457
The Ether Discovery, - - - - - - - - - 458
American Medical Productions and the English Critics, - 458
Patent Medicines. - - - - - - - - - - 459

